# Methotrexate Reduced TNF Bioactivity in Rheumatoid Arthritis Patients Treated with Infliximab

**DOI:** 10.1155/2017/3708250

**Published:** 2017-03-02

**Authors:** Delphine Dénarié, Mélanie Rinaudo-Gaujous, Thierry Thomas, Stéphane Paul, Hubert Marotte

**Affiliations:** ^1^Department of Rheumatology, CHU Saint-Etienne, Saint-Etienne, France; ^2^Immunology and Immunomonitoring Department, CHU de Saint-Etienne, Saint-Etienne, France; ^3^INSERM 1059/SAINBIOSE, Université Jean Monet, Université de Lyon, Saint-Etienne, France

## Abstract

*Objectives.* To evaluate methotrexate effect on tumor necrosis factor (TNF) alpha bioactivity during infliximab (IFX) therapy in rheumatoid arthritis (RA) patients and to correlate TNF bioactivity with antibody towards IFX (ATI) development and RA clinical response.* Materials and Methods.* Thirty-nine active women RA patients despite conventional synthetic disease modifying antirheumatic drugs (csDMARDs) requiring IFX therapy were enrolled, and clinical data and blood samples were recorded at baseline (W0) and at 6 weeks (W6), W22, and W54 of IFX treatment. TNF bioactivity as well as IFX trough and ATI concentrations were assessed on blood samples.* Results.* TNF bioactivity decreased from W0 to W54 with a large range from W22 at the time of ATI detection. From W22, TNF bioactivity was lower in presence of methotrexate as csDMARD compared to other csDMARDs. IFX trough concentration increased from W0 to W54 with a large range from W22, similarly to TNF bioactivity. Methotrexate therapy prevented ATI presence at W22 and reduced TNF bioactivity compared to other csDMARDs (*p* = 0.002).* Conclusion.* This suggests that methotrexate plays a key role in TNF bioactivity and against ATI development.

## 1. Introduction

Since last two decades, management of rheumatoid arthritis (RA) improved strongly RA prognosis due to tight control management and large availability of biological disease modifying antirheumatic drugs (bDMARDs). Infliximab (IFX, Remicade®) is a human-murine chimerical monoclonal IgG antibody targeting tumor necrosis factor (TNF) alpha. IFX was approved to treat RA [1] and other inflammatory diseases after inadequate response to conventional synthetic (cs) DMARDs [2]. Unfortunately clinical improvement is heterogeneous with primary or secondary therapy failure [3]. Many predictors for clinical response were already reported, but none of them are daily used [4] at the time of the “personalised medicine” [5]. Among them, we previously described that high TNF bioactivity was a predictor for a good clinical response to IFX therapy [6]. Detection of antibody towards IFX (ATI) could explain immunoallergic reactions, paradoxical effect, or lack of response to IFX [7]. However, part of the lack of response to IFX could be explained by monitoring IFX trough and ATI concentrations [8]. Since developments of commercial kits for IFX concentration and ATI detection are available in the daily practice, interest of the monitoring of IFX trough and ATI concentrations is growing. Moreover, impact of this monitoring was already investigated to improve management of inflammatory bowel disease (IBD) [9, 10]. Furthermore, TNF bioactivity was mainly driven by IFX trough concentration with some impact of ATI concentration [11]. So, we explored in RA patients the TNF bioactivity before and at various time points after the IFX therapy beginning and correlated it with IFX trough concentration, development of ATI, and clinical response in RA patients.

## 2. Materials and Methods

### 2.1. Patients

Thirty-nine women RA patients with active disease despite csDMARDs and naïve to bDMARDs were enrolled as previously described [12]. All patients gave informed consent. Patients received IFX therapy at 3 mg/kg per infusion at weeks (W) 0, 2, and 6 and then every 8 weeks in combination with csDMARD. Before each infusion, a clinical joint assessment with erythrocyte sedimentation rate (ESR) and C-reactive protein (CRP) determination was performed with DAS28(ESR) calculation. The clinical response was defined according to the criteria of the EULAR [13]. Blood samples were collected before IFX infusion at W0, W6, W22, and W54 and sera were stored at −80°C until used.

Anti-CCP2 and rheumatoid factor (RF) were measured by ELIA method on ImmunoCap 250 (Phadia, Thermo Fisher Scientific, Uppsala, Sweden). Anti-CCP2 was considered to be positive at a cut-off value of 10 U/mL and RF IgM at 3.5 IU/mL as recommended by the manufacturer.

### 2.2. Cell-Based Bioassay for TNF Bioactivity and IFX Trough and ATI Concentration Determination

A functional assay to assess TNF bioactivity was adapted from our previously study [11, 14] by using HEK-Dual TNF Cells (InvivoGen, San Diego, CA). Since sera of patients were not able alone to activate HEK-Dual TNF Cells, sera of patients were first incubated with exogenous recombinant TNF (10 ng/mL, R&D Systems, Abingdon, UK) with or without exogenous infliximab (5 mg/mL). Then, the mix was deposed in wells with HEK-Dual TNF Cells. These cells allowed the specific study of TNF-induced NF-kB activation by monitoring the activity of secreted embryonic alkaline phosphatase (SEAP) with a SEAP detection reagent QUANTI-BlueTM (InvivoGen). TNF bioactivity was defined by SEAP value obtained by combination of sera and TNF minus SEAP value obtained with combination of sera, TNF, and IFX. IFX trough and ATI concentrations were assessed by ELISA with Lisa Tracker Infliximab® Kit (Theradiag®, Marne-La-Vallee, France) on the same samples. High and low IFX trough concentration were defined with a cut-off at 2 *μ*g/mL. ATI positivity was defined by a concentration higher than 20 ng/mL [9].

### 2.3. Statistical Analysis

Due to low number of patients, data were expressed as median and interquartile range 25%–75% [IQR 25–75] or number (%) and nonparametric tests (Spearman test with coefficient Pearson's (*r*_*s*_), *χ*^2^ test, Wilcoxon test, or Kruskal-Wallis test with Dunn's multiple comparison test, as appropriate) were performed. Statistical analyses were performed using R software.

## 3. Results

### 3.1. RA Patient Characteristics

The main RA characteristics were described previously [12]. All patients were women with a median age at 56.3 years [interquartile range 25%–75%; 46.4–61.3] and a disease duration at 12.2 years [6.4–17.7]. Rheumatoid factor and anti-CCP2 were positive in 82.5% of RA patients (for both). At baseline, RA was active despite csDMARDs with a median DAS28 at 5.2 [4.9–5.6]. Methotrexate was the most csDMARDs used in 24 RA patients, whereas leflunomide and hydroxychloroquine were used in 13 and 2 patients, respectively. Thirty patients (77.5%) received corticosteroid at baseline with a median dose at 10 mg/day [5–12]. Current smoking was observed in 12.5%.

### 3.2. TNF Bioactivity during IFX Therapy

**Figure 1:**
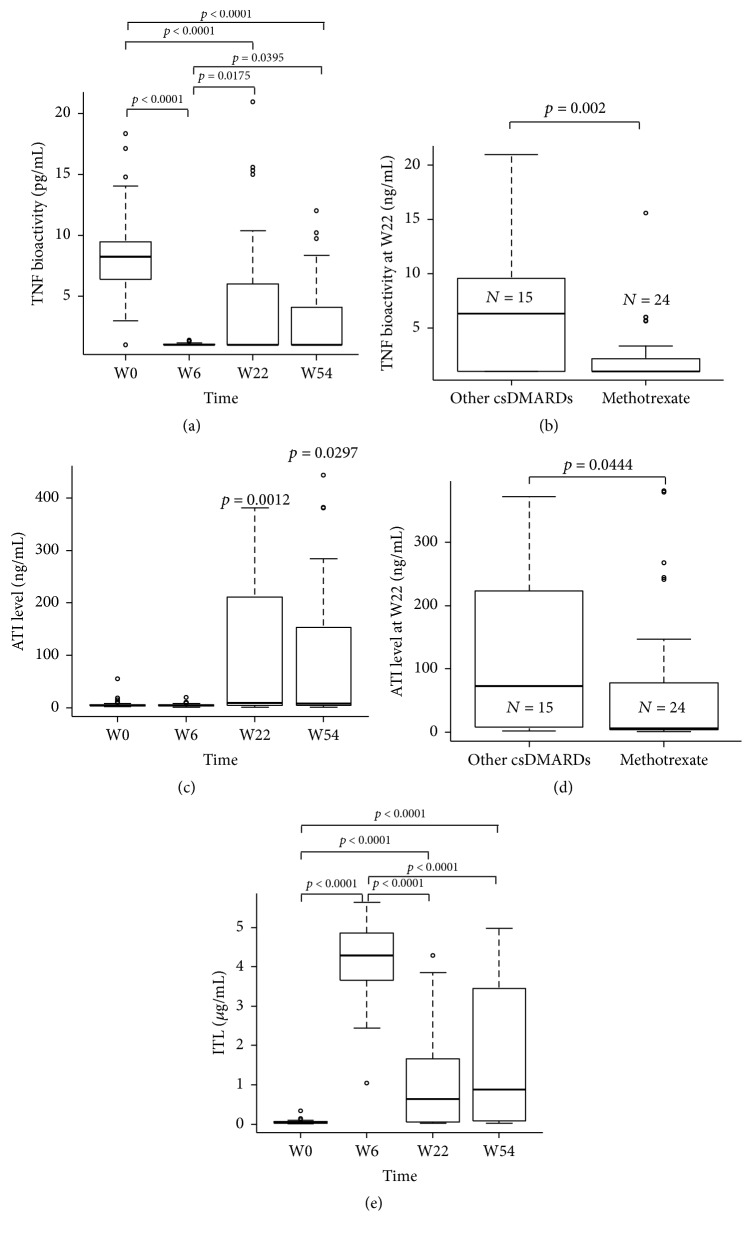
Evolution of TNF bioactivity, IFX trough concentration, ATI concentration during IFX treatment, distribution of TNF bioactivity according to EULAR response, and effect of methotrexate on ATI concentration and TNF bioactivity. TNF bioactivity (a) decreased strongly at W6 with a slight increase afterwards. TNF bioactivity was lower in patients with methotrexate compared to patients with other csDMARDs (b). ATI (c) were detected at W22 and W54 and ATI concentration was lower in patients with methotrexate compared to patients with another csDMARDs (e). IFX trough concentration (d) strongly increased at W6 and then slightly decreased afterwards. The box plots show the median values and the first and third quartiles at each time. The T bars represent the rest of the data with a maximum of 1.5 times the interquartile range. Circles represent values lower or higher than 1.5 times the interquartile range. TNF: tumor necrosis factor; ATI: antibodies towards IFX; W: week; csDMARDs: conventional synthetic disease modifying antirheumatic drugs.

TNF bioactivity strongly correlated with DAS28 when pooling all time points (*r*_*s*_ = 0.371; *p* < 0.0001). As expected, TNF bioactivity was heterogeneous during IFX therapy (Kruskal-Wallis test at 56.4; *p* < 0.0001). High TNF bioactivity was observed at W0 (8.20 ng/mL [6.35–9.46]) and was strongly reduced at W6 (1.00 ng/mL [1.00–1.04]; *p* < 0.0001), further to the IFX loading doses (Figure 1(a)). Then, TNF bioactivity range increased at W22 and W54 (1.00 ng/mL [1.00–6.01] and 1.00 ng/mL [1.00–4.04]; *p* = 0.0395 and *p* = 0.00175, resp., Figure 1(a)). At W22, TNF bioactivity correlated with DAS28 (*r*_*s*_ = 0.38; *p* = 0.024; data not shown). So TNF bioactivity could explain heterogeneity of clinical response to IFX.

### 3.3. Factors Impacting TNF Bioactivity and Clinical Response Heterogeneity

TNF bioactivity at W22 was not explained by baseline clinical parameters including body mass index, smoking status, or baseline corticosteroid doses. However, in RA patients with methotrexate therapy, TNF bioactivity at W22 was lower than that in patients treated with other csDMARDs (Wilcoxon rank sum test *p* = 0.002; Figure 1(b)). So, to explain TNF bioactivity heterogeneity at W22 and W54, we then assessed IFX trough and ATI concentrations in the same blood samples. ATI were detected from W22 (41%; *n* = 16; *p* = 0.0012) to W54 (37%; *n* = 7; *p* = 0.0297; Figure 1(c)). Then, we explored factors explaining ATI development heterogeneity. Methotrexate therapy was associated with absence of ATI development at W22 compared to other csDMARDs (leflunomide or hydroxychloroquine) (*χ*^2^ = 6.13; *p* = 0.0133). Furthermore, ATI concentration was lower in case of methotrexate as csDMARDs compared to leflunomide or hydroxychloroquine (*p* = 0.0444; Figure 1(d)). As expected, IFX trough concentration was heterogeneous during IFX treatment (Kruskal-Wallis test at 56.4; *p* < 0.0001). Median IFX trough concentration increased from 0.04 *μ*g/mL [0.03–0.07] at W0 to 0.88 *μ*g/mL [0.08–3.45] at W54 (*p* < 0.0001; Figure 1(e)). IFX trough concentration strongly increased from W0 to W6 with a small range (4.25 *μ*g/mL [3.65–4.73]; *p* < 0.0001). Then IFX trough concentration decreased with a higher range at W22 (0.63 *μ*g/mL [0.06–1.63]; *p* < 0.0001) and at W54 (0.88 *μ*g/mL [0.08–3.45]; *p* < 0.0001). At W22 and W54, ATI were detected only in RA patients with low IFX trough concentration (*r*_*s*_ = −0.81; *p* < 0.0001 and *r*_*s*_ = −0.56; *p* = 0.002; data not shown).

### 3.4. TNF Bioactivity according to IFX Trough Concentration and ATI Threshold

**Figure 2:**
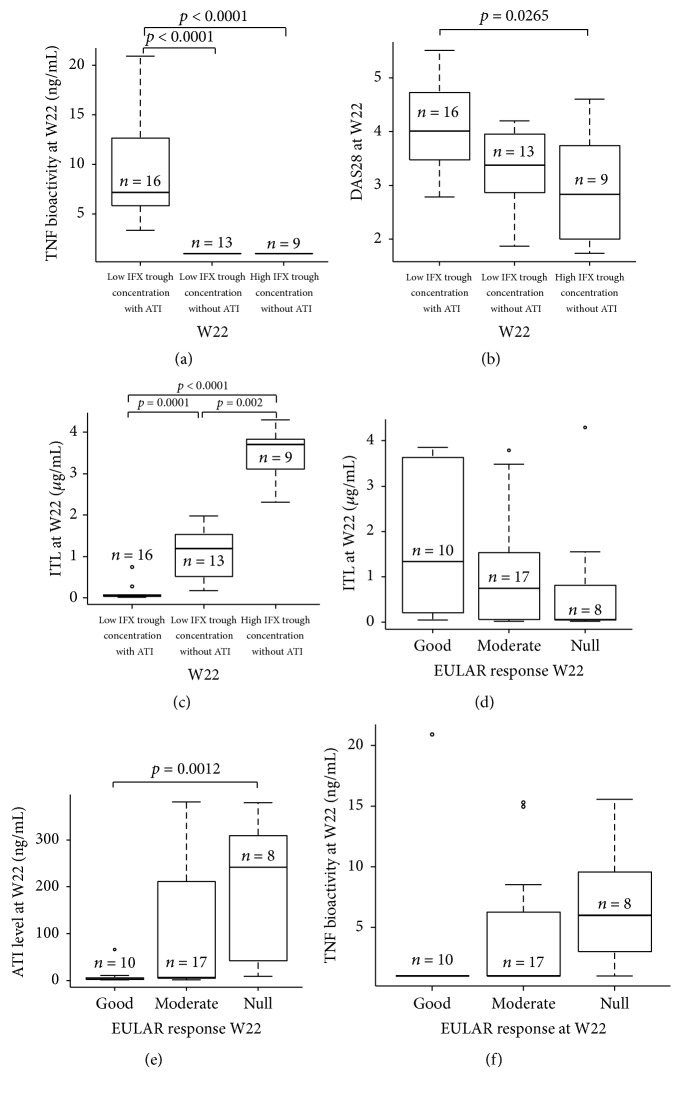
TNF bioactivity, DAS28, and IFX trough concentration at W22 in function of presence of IFX with or without ATI at W22 and IFX trough concentration and ATI at W22 and clinical response at W22. High TNF bioactivity was observed in the group “Low IFX trough concentration with ATI,” but not in the groups “Low IFX trough concentration without ATI” and “High IFX trough concentration without ATI” ((a) Kruskal-Wallis rank sum test; *p* < 0.0001). DAS28 was higher in the “Low IFX trough concentration with ATI” group compared to the two others ((b) Kruskal-Wallis rank sum test; *p* = 0.0266). IFX trough concentration at W22 was heterogeneous in the three groups ((c) Kruskal-Wallis rank sum test; *p* < 0.0001) with highest IFX trough concentration in the group “High IFX trough concentration without ATI.” Furthermore, IFX trough concentration was higher in the group “Low IFX trough concentration without ATI” than in the group “Low IFX trough concentration with ATI” ((c) *p* < 0.0001). IFX trough concentration was higher in the group “High IFX trough concentration without ATI” than in the group “Low IFX trough concentration without ATI” ((c) *p* = 0.0002). A trend for high IFX trough concentration and good EULAR response was observed at W22 ((d) Kruskal-Wallis rank sum test *p* = 0.1526). Oppositely, ATI concentration was not detectable in good response and high in Null response at W22 ((e) Kruskal-Wallis rank sum test *p* = 0.0047). At W22, a trend between TNF bioactivity and EULAR response (f). TNF bioactivity was not detectable in good response and high in Null response at W22 (Kruskal-Wallis rank sum test; NS). The box plots show the median values and the first and third quartiles at each time. The T bars represent the rest of the data with a maximum of 1.5 times the interquartile range. Circles represent values lower or higher than 1.5 times the interquartile range. TNF: tumor necrosis factor; ATI: antibodies towards IFX; W: week; Low ITL: ITL lower than 2 *μ*g/mL; High IFX trough concentration: infliximab trough concentration higher than 2 *μ*g/mL; presence or absence of ATI was determined with a cut-off at 20 ng/mL.

Due to negative correlation between IFX trough concentration and ATI concentration, we explored TNF bioactivity in 3 groups according to IFX trough concentration and ATI positivity at W22: (1) Low IFX trough concentration without ATI, (2) Low IFX trough concentration with ATI, and (3) High IFX trough concentration without ATI. TNF bioactivity was heterogeneous in the 3 groups (Kruskal-Wallis rank sum test *p* < 0.0001). High TNF bioactivity was observed only in the group “Low IFX trough concentration with ATI” (*p* < 0.0001; Figure 2(a)). Similarly to TNF bioactivity, DAS28 was heterogeneous in these 3 groups (Kruskal-Wallis rank sum test *p* = 0.0266) with highest level in the “Low IFX trough concentration with ATI” group compared to the “High IFX trough concentration without ATI” group (*p* = 0.0265; Figure 2(b)). To investigate the discrepancy of absence of TNF bioactivity in the “Low IFX trough concentration without ATI,” we checked IFX trough concentration in these 3 groups (Figure 2(c)). Again, IFX trough concentration was heterogeneous in these 3 groups (Kruskal-Wallis rank sum test; *p* < 0.0001). In the group “Low IFX trough concentration without ATI,” IFX trough concentration was higher (1.19 ng/mL [0.59–1.54]) than in the group “Low IFX trough concentration with ATI” (0.06 ng/mL [0.04–0.07]; *p* < 0.0001) explaining absence of TNF bioactivity in the “Low IFX trough concentration without ATI” group.

### 3.5. Correlation between Clinical Response and IFX Trough or ATI Concentrations

IFX trough concentration (Figure 2(d); *p* = 0.1526) and ATI concentration (Kruskal-Wallis rank sum test *p* = 0.0047; Figure 2(e)) were different according to EULAR response at W22. However, a trend was observed between the TNF bioactivity and EULAR response at W22 (Figure 2(f); *p* = 0.07). At W22, DAS28 correlated negatively with IFX trough concentration and positively with ATI concentration (*r*_*s*_ = −0.36; *p* = 0.032 and *r*_*s*_ = 0.6; *p* = 0.0001; resp.).

## 4. Discussion

At the time of personalised medicine [5], only few data were available for monitoring RA patients with IFX trough concentration and/or ATI dosage to improve RA therapy strategy. This is critical since commercial kits for IFX and ATI monitoring are available in daily practice. These dosages already provided some interests in IBD therapeutic management [9, 10]. Some years ago, TNF bioactivity was already described as a biomarker to predict clinical response to IFX [6]. So, the purpose of this study was to explore variation of TNF bioactivity during IFX therapy and to investigate correlation between TNF bioactivity or IFX trough or ATI concentrations and clinical response. As expected, a strong reduction of TNF bioactivity was observed at W6 with some heterogeneity later [6]. This correlated strongly with IFX trough concentration which increased from baseline to W6 [15]. Furthermore, range of TNF bioactivity appeared lower at W6 compared to range of IFX trough concentration. This was not surprising due to high IFX trough concentration at W6 [11]. In the later time points, heterogeneity of TNF bioactivity was connected to heterogeneity of IFX trough and ATI concentrations. Since our results confirmed association between low IFX trough concentration and ATI presence, IFX trough concentration heterogeneity and TNF bioactivity could be in part related to ATI development [16].

We then explored the heterogeneity of TNF bioactivity at W22. At baseline, the only factor influencing TNF bioactivity at W22 was presence of methotrexate as csDMARDs. By preventing ATI development, RA patients with methotrexate therapy obtained higher IFX trough concentration with a lower TNF bioactivity than other RA patients. This heterogeneity of TNF bioactivity as for IFX trough concentration could explain only some part of clinical response heterogeneity as previously described in RA [8, 16]. Despite a large consensus between IFX trough concentration and good clinical response [9, 15, 17], data on ATI interest remained controversial in part due to heterogeneity of tests used for their determinations [18]. According to ELISA test used in our study for ATI determination, we confirmed that ATI were detectable only in patients with low IFX trough concentration [16]. Development of ATI seemed more frequent in RA than in spondyloarthritis (SpA) [16] with 40% in our study consistently with previous reports at the same time point [16, 19]. Furthermore, we confirmed association between presence of ATI and a lower EULAR response [15, 19], confirming a neutralizing role on IFX [11].

Preventing effect of methotrexate on ATI development was not extensively analyzed. In ATTRACT study [15], all RA patients received methotrexate with a dose at least 12.5 mg per week. This was due to early study showing higher IFX trough concentration in presence of methotrexate [17]. Two mechanisms were at this time proposed to explain this association between methotrexate and high IFX trough concentration [17]. First, methotrexate by himself is efficient on RA activity and could reduce amount of IFX binding to reduce inflammation as suggested recently in RA [20] or in IBD [10]. Second, MTX could decrease immunogenicity against IFX. This last mechanism remained controversial [16, 19]. However, in RA patients with ATI, methotrexate therapy allowed an increasing of IFX treatment duration [21], suggesting a beneficial effect of methotrexate on ATI concentration or function. Contrarily to methotrexate, glucocorticoid (presence or dose) was not found to prevent development of ATI in this cohort, as previously described [16]. Additionally, this is the first report suggesting a better effect of methotrexate compared to two other csDMARDs (leflunomide and hydroxychloroquine) in prevention of ATI development. Despite methotrexate, only azathioprine demonstrated a benefit to prevent ATI development in IBD [22, 23].

Interestingly, in the group “Low IFX trough concentration without ATI” no TNF bioactivity was observed, whereas TNF bioactivity was observed in the group “Low IFX trough concentration with ATI.” Explanation of this putative discrepancy between TNF bioactivity in both groups was due to higher IFX trough concentration in absence of ATI. This also explained heterogeneity of DAS28 at W22 in these three groups. The association between ATI and low TNF bioactivity demonstrates that the presence of ATI is preventing endogenous TNF neutralization by infliximab [11].

Our data have some weakness with 39 patients included at baseline. We failed to observe correlation between low IFX trough concentration and development of ATI probably due to absence of blood samples around 3 months of IFX therapy. The paradox results between TNF bioactivity and low IFX trough concentration in presence or not of ATI will require further investigations.

To conclude, we described a major role of methotrexate to reduce ATI development, to reduce TNF bioactivity, and to improve DAS28 compared to other csDMARDs. Our result confirmed mechanisms of lack of IFX efficacy by ATI development with impact on TNF bioactivity.
